# Editorial: Fruit science: Physiological changes and genetic regulations

**DOI:** 10.3389/fpls.2023.1178844

**Published:** 2023-03-31

**Authors:** Hua Huang, Ling Wang

**Affiliations:** ^1^ Institute of Fruit Tree Research, Guangdong Academy of Agricultural Sciences, Key Laboratory of South Subtropical Fruit Biology and Genetic Resource Utilization, Ministry of Agriculture and Rural Affairs, Guangdong Provincial Key Laboratory of Tropical and Subtropical Fruit Tree Research, Guangzhou, China; ^2^ Sericultural & Agri-Food Research Institute Guangdong Academy of Agricultural Sciences, Key Laboratory of Functional Foods, Ministry of Agriculture and Rural Affairs, Key Laboratory of Agricultural Products Processing, Guangzhou, China

**Keywords:** fruit color, postharvest senescence, volatile compounds, pathogen infection, genetic

Fruits are among the main organs of plants, concealing seeds during the mature stage, which also provide a valuable food resource for the diets of humans and other animals. The development and ripening of fruit involves a series of structural, physiological, and genetic regulatory processes (Giovannoni, 2004; Seymour et al., 2013). Typical changes in fruit ripening include color development associated with the production of pigment compounds, such as anthocyanins, chlorophyll, and carotenoids, along with the production of aroma volatiles, changes in texture associated with cell wall and cuticle modification, and differences in the incidence of pathogen infection (Chen et al., 2021). In addition, mature fruit is sensitive to environmental factors and prone to post-harvest senescence. Consequently, appropriate post-harvest management is of particular importance with respect to the prevention of rapid quality deterioration and pathogen invasion, thereby contributing to a prolongation of shelf-life. This edition was conceived with the aim of updating our current understanding of fruit ripening by presenting the findings of recent research that has focused on fruit quality development and the measures adopted to enhance post-harvest preservation.

The color and aroma volatiles of fruit differ according to differences in the secondary metabolites that accumulate in the peel and flesh of different fruits. Li et al. have compared the pigmentation of pulp and peel during the development of two varieties of pitaya fruit, *Hylocereus undatus* (white pulp) and *Hylocereus polyrhizus* (red pulp), based on a combination of transcriptome and biochemical analyses. This study demonstrated that color development in purple-red pitaya fruit was primarily associated with the biosynthesis of betalains, which accumulate in both varieties as red peel, although to a considerably less extent in the white-pulped *H. undatus*. The authors also found that the ripening of pitaya fruit is regulated by coordinated hormonal activity, particularly *via* the signal transduction of auxin and ABA. In addition to the attractive fruit color, the accumulation of aroma compounds also plays important roles in quality development. In this regard, Yang et al. detected 38 volatile compounds in the fruit of a ‘Fuji’ × ‘Cripps Pink’ apple population, and also screened 87 quantitative trait loci for 15 volatile compounds, based on a combination of aroma phenotypic data and a constructed genetic linkage map. They accordingly identified a candidate gene, *MdAAT6*, involved in regulating the production of esters, which was verified by ectopic expression in tomato plants. Currently, a diverse range of transcription factors and epigenetic modifications have been reported to be associated with regulation of the fruit-ripening process (Li et al., 2022), and thus comprehensive studies based on the combined analyses of biochemical, plant hormone, and genetic regulation would contribute to gaining a better understanding of the development of fruit quality, and thereby enhance the efficacy of the breeding process.

Fruit ripening is influenced to a large extent by the maintenance of reactive oxygen species (ROS) homeostasis in cells, and in this regard, Hou et al. have identified 78 genes that encode Class III peroxidases (AcPRXs), which play roles in the regulation of ROS levels. Transcriptomic analysis revealed that gene of *AcPRXs* were associated with the negative regulation of the internal browning in post-harvest pineapple fruit. The authors found that soaking in ascorbic acid could inhibit the excessive production of ROS and polyphenol oxidase (PPO) activity, thereby alleviating the occurrence of internal flesh browning in these fruits. These findings accordingly highlight the importance of identifying appropriate strategies that can be adopted to prevent, or at least delay the rapid senescence of fruit after harvest. Zeng et al. evaluated the effects of exogenous naringin treatment designed to enhance the disease resistance of citrus fruit. On the basis of transcriptomic analysis, they detected the significant upregulation of 12 genes in the phenylpropanoid and flavonoid biosynthesis pathways, and by performing metabolomic analysis, they detected a total of 325 flavonoids, among which increases in auraptene, butin, naringenin, and luteolin derivatives were promoted in response to naringin treatment. In addition to changes in metabolites and their regulatory genes, transcription factors of MYC and WRKY family may also play important roles in the disease resistance responses of plants, and in this respect, natural biologically induced disease resistance in fruit is seen as a further green strategy that could be adopted to prevent or minimize pathogen invasion. Li et al. have reported an elicitor protein, phosphopentomutase, identified in *Bacillus velezensis* LJ02 (BvEP), which was demonstrated to enhance the resistance of tomato to *Botrytis cinerea*. They found that BvEP promoted an up-regulated expression of genes associated with PAMP-triggered immunity (PTI), effector-triggered immunity (ETI), and systemic acquired resistance (SAR) as defensive measures to counteract the invasion of *B. cinerea*. In addition, BvEP was observed to promote increases in the accumulation of ROS and enhanced the activity of antioxidant enzymes in tomato fruit.

The articles presented in this edition dedicated to the physiological changes and genetic regulation of fruit ripening have contributed to elucidating the biochemical changes associated with color and volatile development, and their regulation, based on genetic and multiple omics analyses. In addition, they have also examined post-harvest treatments adopted with the aim of delaying fruit senescence, as well as promoting resistance to pathogen invasion. Collectively, they provide important insights that highlight the complexity of the regulatory processes that underlie the observed changes of fruit quality throughout the entire life of fruits, from development to ripening, as well as during post-harvest senescence. Despite these valuable findings, however, further in-depth investigations, based on a combination of structural, biochemical, physiological, and molecular/genetic analyses, are required to gain a more comprehensive understanding of these processes. The findings of such studies will make an important contribution to facilitating the breeding of high-quality fruit varieties, as well as guiding the development of efficient post-harvest preservation strategies for different varieties of fruit.

## Author contributions

All authors listed have made a substantial, direct, and intellectual contribution to the work and approved it for publication.
